# Costs of Treatment after Renal Transplantation: Is it Worth to Pay More?

**Published:** 2014

**Authors:** Jamshid Salamzadeh, Naghmeh Foroutan, Hamid Reza Jamshidi, Hamid Reza Rasekh, Ali Rajabzadeh Gatari, Arash Foroutan, Mohsen Nafar

**Affiliations:** a Department of Pharmacoeconomics and Pharmaceutical Management, School of Pharmacy, Shahid Beheshti University of Medical Sciences, Tehran, Iran.; b Tarbiat Modares University.; c School of Pharmacy, Tehran University of Medical Sciences.; d Department of Kidney Transplantation, Urology Nephrology Research Center (UNRC), Shahid Labbafinejad Medical Center, Shahid Beheshti University of Medical Sciences, Tehran, Iran.

**Keywords:** Cost, Renal transplantation therapy, Insurance, Policy making, Budget

## Abstract

The primary aim of the study was to estimate costs of treatment for the first year after renal transplantation from the perspective of health insurance organizations in Iran.

An Excel-based and a Monte Carlo model were developed to determine the treatment costs of current clinical practice in renal transplantation therapy (RTT). Inputs were derived from Ministry of Health and insurance organizations database, hospital and pharmacy records, clinical trials and local and international literature. According to the model, there were almost 17,000 patients receiving RTT in Iran, out of which about 2,200 patients underwent the operation within the study year (2011 - 2012; n = 2,200)

The estimated first year total treatment cost after renal transplantation was almost $14,000,000. These costs corresponded to annual total cost per patient of almost $6500 for the payers.

Renal transplantation therapy is almost fully reimbursed by government in Iran. However, regarding new expensive medicines, cost of medical expenditure is rapidly growing and becoming quite unaffordable for the government; therefore, out-of-pocket (OOP) payments are dramatically increasing over time. In order to improve reimbursement policy making under pressure of current budget constraints, the present study is providing decision makers with practical tools make it possible for them to easily compare budgetary impact of the current therapy strategy with the future financial consequences of purchasing newly proposed medicines. In other words having estimation of the current budget spending on RTT would help policy makers in making efficient resource allocation and decrease quite high OOP expenditures.

## Introduction

The number of patients with end-stage renal disease (ESRD) is increasing worldwide at a rate of almost 7% to 8% per year (1). Renal transplantation therapy (RTT) has been considered a cost-effective alternative therapy compare other renal replacement therapies (RRT) such as hemodialysis or peritoneal dialysis in patients suffering from end-stage renal disease (ESRD) (2) Prevalence and incidence of ESRD in Iran is almost 357 per million population (pmp) and 66 pmp, respectively (3, 4). From March 2011 to March 2012, there were almost 17,000 patients receiving RTT in Iran, out of which about 2,200 patients had renal transplantation.

In recent years, because of economic crises, policy makers have faced much more difficulties in allocating limited available budget to several diseases. Notwithstanding renal replacement therapies (RRT) - transplantation and hemodialysis-have been grouped as special diseases and receive subsidy along with full reimbursement coverage in Iran, out-of-pocket (OOP) expenditure is considerable regarding both of the mentioned therapies. 

Renal transplantation (RT) has a quite long history in Iran. The first surgery in the Middle East region was performed in 1967 in Shiraz, south of Iran. Since then, the number of candidate patients for receiving renal transplantation increased; however, dialysis was still the treatment of choice due to the fact that patients used to go to European countries for the transplantation procedure with a public fund; thus, it was costly for Ministry of Health and also there was a long transplant waiting list for patients. Finally, in 1985, the government decided to provide patients with renal transplantation operation inside the country (5, 6).

Considering Article 29 of Iranian Constitution, according to which accessibility and affordability of health services for the entire population are a policy focus of the Iranian Government (7) there are special facilities and reimbursement processes provided by Ministry of Health and insurance organizations which make RTT and dialysis more affordable and also accessible for eligible patients from different socioeconomic status. There are several governmental and quasi-government organizations engaged in ESRD issue: a) Ministry of Health (MOH) as the main sponsor, b) Social Security Organization (SSO), c) Medical Service Insurance Organization (MSIO), d) Armed Forces Medical Service Organization (AFMSO), e) Imam Khomeini Relief Foundation (IKRF), and f) Special Organizations such as Ministry of Welfare and Social Security, oil companies, radio and television broadcasters, banks and non-governmental organizations (NGOs) (7,8).

Nowadays, regarding new expensive medicines and interventions, cost of RRT medical expenditureis rapidly growing and becoming quite unaffordable for the government; therefore, OOP paymentsare dramatically increasing over time.

In order to improve reimbursement decision making under pressure of current budget constraints, there is an essential need to provide insurance policy makers with effective financial budgeting tools and thus allocate the budget in a way that the trade-off between effectiveness and staying within the budget is always sustained in terms of therapeutic guidelines. 

In the present study, current treatment cost of renal transplantation therapy was calculated for Iranian insurance organizations in order to provide them with practical tools, make it possible for them to have an estimation of financial impact of current practice in RTT, especially in comparison with other RRTs (mainly hemodialysis), and also provide them with the chance of simply calculating budgetary impact of purchasing newly introduced cost-effective medicines in RTT to be provided with satisfactory insurance coverage in the future. 

## Experimental

Estimates of patient population and data sources .Population-based incidence data were obtained from a central registry system in the Management Center for Transplantation and Special Diseases (MCTSD), affiliated to Ministry of Health (MOH) (9). Prevalence data were not considered in this study. Tariffs and expenditure data of RTT and dialysis were extracted from insurance organizations` database. Regarding required probabilities, hospital-based registries, local clinical trials and national and international literature were reviewed.

Clinical data were obtained on the following key events: initial hospitalization for transplantation, immunosuppressive drug use, graft failure, acute rejection, delayed graft function, cytomegalovirus (CMV) infection and other important adverse events’ treatment. 

Estimates on in-patient drug use, treatment duration and rate of rehospitalization were based on local standard protocol for RTT and dialysis, SSO and MSIO databases and hospital-based registries. Estimates on out-patient details of the immune suppressive regimen were obtained from Helal -e- Ahmar pharmacy records and SSO database.

According to MCTSD and database of insurance organizations, during the study year (2011- 2012), almost 17,000 patients were receiving RTT, out of which about 2,200 patients (aged between 18-70 years old) had their transplantation operation. There are two main semi-public hospitals in Tehran (capital city) performing renal transplantation which are in charge of almost half of the operations in the country (25% Baghiatollah Hospital and 25% Shahid Labbafinejad Teaching Hospitals). 

*Cost of initial hospitalization for transplantation*

There is a global fixed tariff (GT) for transplantation provided by insurance organizations in both public and private hospitals with no co-payment or franchise for patients (except 10% for hospitalization in some hospitals). According to tariffs in the study year, a fixed amount of almost 39,000,000 IRR ($3,181US) was paid for each pair of operation (recipient and donor) for the whole hospitalization period (5 to 30 days). Total tariff was defined as 650 K, in which K was almost 60,000 IRR in the study year. Some drugs (*e.g*. ganciclovir) are not included in the GF. In some cases, real costs become greater than the compensation and patients should pay the difference as OOP. Considering the average annual number of patients having renal transplantation (2,200 patients), the total budget spent for initial hospitalization for transplantation was almost 86 billion IRR ($7,000,000). 

*Cost of immune suppressive agents*

regarding maintenance therapy (MT) in RTT, the most costly and important immunosuppressive drugs are cyclosporine (CsA), mycophenolate mofetil (MMF) (10), sirolimus (SRL) and tacrolimus. The mean CsA dose is 150 mg/d per patient and is available in three dosages (25, 50, 100 mg oral tablets); only the generic form is included in the insurers` formulary. The CsA cost was acquired from database of insurance organizations and calculated based on proportion of patients normally using different dosages (based on pharmacy records). Tacrolimous was usually used in patients who had their second or more transplantation (almost 10%) and the rest (90%) used CsA as the main immune surpressive agent. Rapamune^®^ (SRL) was only administered in special cases (less than 1%); however, it was not included in the analysis due to the fact that,over the study year, it did not have any insurance coverage (Table 1). 

**Table 1:** Cost of maintenance therapy (immune suppressive agents) according to tariffs as unit price in both IRR and USD (2011- 2012).

**Maintenance Therapy**	**Dosage form**	**Dosage/day/ patient**	**Unit price (IRR)**	**Total cost/day /patient (IRR)**	**Nr. of Eligible patients**	**Duration (D.)**	**Total cost (IRR)**	**Total cost ($USD)**
**Cyclosporine (Generic)**	Cap: 25, 100 ,50 mg	150^†^ mg qd	300, 1200, 1500	5,167,800^‡^	-	365	1,886,247,000	153,854
**MycophenolateMofetil**	Cap: 500 mg	2 g qd	11,000	44,000	2,200	365	35,332,000,000	2,881,892
**Prednisolone**	Tab: 5 mg	5mg/d qd	130	130	2,200	365	104,390,000	8,515
**Tacrolimus**	Tab: 0.5 mg	0.2mg/Kg/d	1,000	28,000	220^§^	365	2,248,400,000	183,393
**Total cost**							39,571,037,000	3,227,654

D.= day; qd= once per day, everyday.

† Almost 60% (more than half) of the patients receiving 150 mg/day qd. The rest, are using 100 mg (5%), 125 mg (10%), 175 mg (10%), 200 mg (10%) and 225 mg (5%) per day (Source: insurance organizations database).

‡Total cost-patient has been calculated based on proportion of patients using different dosages. Number of patients using Tacrolimus has been excluded.

§In average 10% of patients have their second or more transplantation and use Tacrolimus.

Cost of adverse events (AE) treatment major adverse events related to CsA-based regimens during 12 months posttransplantation are mainly acute rejection (18%) (4), delayed graft function (17%) (11), CMV infection (21%), graft failure (10.5%), thrombocytopenia (8%), hyperlipidemia (14%) (12) and hypertension (67%) (13-17) Cost of each adverse event per patient and total cost of adverse events are summarized in Tables 2 and 3.

**Table 2:** Cost of adverse events per patient in RTT using tariffs as unit price.Total cost values are in IRR and USD, Iran (2011-2012).

**Adverse Events/ Treatment**	**Dosage form**	**Dosage/day/ patient**	**Nr. per day**	**Duration (D.)**	**Unit price (IRR)**	**Total cost /patient (IRR)**	**Total cost ($US)**
**CMV**							
**Ganciclovir**	For inj: 500mg	For treatment: 5 mg/Kg q 12 hr until treatment	2	7	250,000	3,500,000	285
**Hospitalization**				7	1,600,000	11,200,000	914
**Acute Rejection**							
**Methylprednisolone**	For inj: 500 mg	250-1000 mg	1	7	200,000	1,400,000	114
**ATG**	Inj: 250mg/5mL	10-20mg/Kg	4	7	550,000	15,400,000	1,256
**Gancyclovir**	For inj: 500mg	For prophylaxy: 5 mg/Kg q 24 hr until hospitalization	1	7	250,000	1,750,000	143
**Hospitalization**				7	1,600,000	11,200,000	914
**Graft Failure**							
**Dialysis**				10 times	743,200	7,432,000	606
**Re- transplantation**					650K^§^	39,000,000	3,181
**Delayed Graft Function**							
**ATG**	Inj: 250mg/5mL	10-20mg/Kg	4	10	550,000	22,000,000	1,794
**Gancyclovir**	For inj: 500mg	For prophylaxy: 5 mg/Kg q 24 hr until hospitalization	1	10	250,000	2,500,000	204
**Dialysis**				3 times	743,200	2,229,600	182
**Infections**							
**Co-trimoxsazole**	Tab: 400/80mg	q.d. –b.i.d.	2	180	210	75,600	6
**Cefazoline**	For Inj: 1g	3g	3	2	6,000	36,000	3
**Nystatin**	Tab: 500,000 U	t.i.d- q.i.d.	4	180	450	324,000	26
**Fluconazole**	Tab: 100mg	100 mg	1	180	1,200	216,000	18
**Hyperlipidemia**							
**Atorvastatin**	Tab: 10, 20, 40 mg	10-40 mg	1	365	1,100	401,500	33
**Hypertension**							
**Amlodipin**	Tab: 5 mg	5-10 mg	1	365	150	54,750	4
**Thrombocytopenia**							
**Plasmapheresis**			-	10 times	1,300,000	13,000,000	1,060
**Total**						131,719,450	10,744

CMV = cytomegalovirus; ATG = Antithymocyte globulin; hr = hour; b.i.d.= twice per day; t.i.d.= three times per day; q.i.d.= four times per day; q.d. = once per day, every day.

§K: 60,000 IRR.

**Table 3:** Total cost of adverse events related to the current practice in RTT according to the standard guidelines, Iran (2011- 2012).

**Adverse Events**	**Probability**	**Unit cost per patient (IRR)** ^ *^	**Prob. Unit cost /patient**	**Total cost (IRR)** (n = 2,200)	**Total Cost ($US)**
**CMV**	0.21	14,700,000	3,087,000	6,791,400,000	553,948
**Other Infections**	1^**^	651,600	651,600	1,433,520,000	116,927
**Delayed Graft Function**	0.17	26,729,600	4,544,032	9,996,870,400	815,405
**Acute Rejection**	0.18	29,750,000	5,355,000	11,781,000,000	960,930
**Hyperlipidemia**	0.14	401,500	56,210	123,662,000	10,087
**Hypertension**	0.67	54,750	36,683	80,701,500	6,583
**Graft Failure**	0.105	46,432,000	4,875,360	10,725,792,000	874,861
**Thrombocytopenia**	0.08	13,000,000	1,040,000	2,288,000,000	186,623
**Total cost**			19,645,885	43,220,945,900	3,525,363

^*^For each adverse event, unit cost per patient has been calculated in Table 2.

^**^Probability equal to 1 means that the medication has been considered for all patient population (100%, n = 2, 200).

Regarding CMV infection, ganciclovir was administered over a 7-day hospitalization with an average treatment dose of 5 mg/Kg every 12 hours. In the case of acute rejection episodes, Methylprednisolone (daily boluses of 250- 1000 mg), antithymocyte globulin (ATG) with a daily dose of 10- 20 mg/Kg and ganciclovir for CMV infection prophylaxis (5 mg/Kg every 24 hour over hospitalization duration) are administered in renal transplant recipients. ATG with quite similar dosage is also used in patients experiencing delayed graft function. Other adverse events along with their treatment details are explained in Table 2. There are also miscellaneous medicines usually used after transplantation which are listed in Table 4.

**Table 4:** Total cost of miscellaneous medicinesas posttrans plantation medication according to local standard guidelines in RTT, Iran (2011- 2012).

**Miscellaneous medicines**	**Dosage form**	**Dosage/patient**	**Nr. per day**	**Duration (D.)**	**Unit price (IRR)**	**Total cost /patient (IRR)**	** Nr. of eligible patients **	**Total cost (IRR)**	**Total cost ($US)**
**Calcitriol (Rocaltrol®)**	Cap: 0.25 mcg	q.d.	1	365	2300	839,500	2,200	1,846,900,000	150,644
**Ferrus Sulphate**	Tab: 50 mg	q.d.	1	365	55	20,075	2,200	44,165,000	3,602
**A.S.A**	Tab: 80 mg	q.d.	1	365	83	30,295	2,200	66,649,000	5,436
**Ranitidine**	Tab: 150 mg	h.s.	1	365	55	20,075	2,200	44,165,000	3,602
**Calcium Carbonate**	Tab: 500 mg	q.d.	1	365	155	56,575	2,200	124,465,000	10,152
**Total Cost **								2,126,344,000	173,438

D. = day; A.S.A= acetylsalicylic acid, Aspirin; q.d. = everyday, once per day; h.s. = at bed time

*Analysis*

 The model was constructed using Microsoft Excel^®^ 2010. The total cost included cost of initial hospitalization for transplantation, cost of immunosuppressive agents, cost of adverse events and miscellaneous medicines. Total cost was expressed in both Iranian Rials (IRR) and US dollars ($USD). According to the official exchange price in the study year, one USD was equal to 12,260 IRR. 

*Monte-Carlo simulation*

 In order to test the reliability of each individual AE probability used as the model input, a Monte-Carlo model was run, to simulate a random sampling of 2,200 patients out of total population.

The idea of the Monte-Carlo analysis is the generation of large number of (*e.g.* 100 - 1000) synthetic datasets that are similar to the experimental dataset, but with different distributed noise. Each of these new datasets is analyzed, and the distributions are stored. The resulting set of distributions can then be studied, point by point, and the mean and probability contours could be calculated (18, 19).

## Results

In the present study, treatment cost of renal transplantation was calculated for a 1-year time horizon (first year) from the perspective of insurance organizations. 

**Figure 1:**
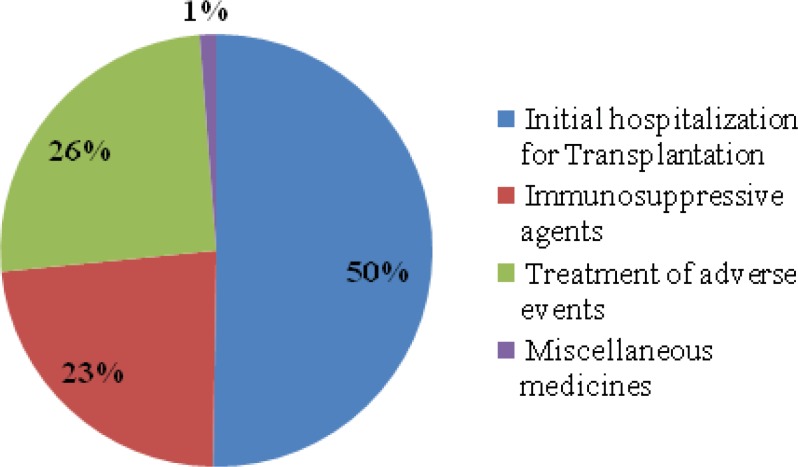
Total treatment cost components of renal transplantation therapy, Iran (2011- 2012).

According to the model, almost 2,200 patients with ESRD had their renal transplantation in the study year. The estimated expected year1treatment cost of RTT with the current standard therapy strategy was almost 171 billion IRR ($14,000,000) for insurance organizations, which was almost 78 million IRR ($ 6,500) per patient. These figures included cost of initial hospitalization for transplantation (50% of total costs), immunosuppressive agents (23% of total costs) and cost of adverse events (26% of total costs) and miscellaneous drugs (1% of total costs) (Table 5, Figure 1). 

**Table 5:** Total treatment cost of renal transplantation therapy for insurance organizations in Iran (2011-2012).

**Treatment Cost of RTT**	**Values (IRR)**	**Values ($US)**
**Initial hospitalization for Transplantation**	85,800,000,000	6,998,369
**Immunosuppressive agents**	39,571,037,000	3,227,654
**Treatment of adverse events**	43,220,945,900	3,525,363
**Miscellaneous medicines**	2,126,344,000	173,438
**Total Costs**	170,718,326,900	13,924,823
**Total Cost/patient**	77,599,240	6,329

Regarding Monte-Carlo simulation, in essence, the model allows building up a probability distribution function, and thus studying how much the noise of the data is translated into the distribution. In the current study, the best-fit distribution represents the 'true' data well enough, so that the effects of noise on the simulated data and the real data are quite the same (18, 19).

According to Table 3, the average cost of AEs per patient calculated based on probabilities extracted from the literature was quite similar to the average cost of AEs per patient calculated by Monte Carlo model throughout several repeated simulations (19,600,000 IRR; $1,600 vs. 19 – 20 million IRR ; $1550- 1630). It shows that the probabilities are as much reliable as if came from a random sampling.

## Discussion

At present, Iran has one of the most successful transplantation programs in the Middle East (6). In Iran, renal transplantation is grouped as a special disease which is paid by different organizations. MOH as the main sponsor along with health insurance organizations covers almost all medicines and medical services included in the standard guidelines approved to be used in RTT. 

In order to improve quality of care in terms of patient and graft survival, policy makers of insurance organizations should always make choice between newly introduced drugs, which are quite expensive, and the current alternatives (5) that is exactly when economic and financial evaluations could play an essential role in making reimbursement decisions (20). 

In the present study, treatment cost of renal transplantation during the first year was calculated to provide insurance policy makers with an estimation of current practice expenses and compare it with future financial consequences of newly proposed medicines have been applied to be added to their formulary (budget impact analysis) (21). Based on the results of this study, considering 17,000 patients receiving RTT, out of which about 2,200 patients had their renal transplantation operation during the study year, the estimated 1-year therapy cost of renal transplantation was almost 78 million IRR ($ 6, 500) per patient from Mar. 2011 to Mar. 2012. More than half of this expense was related to the initial hospitalization for transplantation (Figure 1), highlighting the importance of improving preventive interventions such as using more potent and effective immunosuppressive agents to have fewer cases of graft failure and re-transplantation.

Further to the present study, the first budget impact analysis has been conducted in Iran on applying mTOR inhibitors as immunosuppressive medication in replace to Ciclosporine (22).

*Policy making recommendationsRenal transplantation therapy versus hemodialysis*

According to the literature, in ESRD, costs of renal replacement therapy (RRT) by hemodialysis are far greater than cost of renal transplantation therapy (23, 24). According to the results of the present study, in Iran, only considering hospital-based hemodialysis tariff and required important medications such as erythropoietin and calcitriol, without including any adverse event expenses, 1-year therapy cost per patient would be almost IRR 137 million ($11,000), which is about 2 times greater than cost of RTT per patient (78 million IRR; $ 6,500) (Table 6). 

**Table 6:** Annual cost of dialysis in case all patients receive hemodialysis rather than renal transplantation, Iran (2011- 2012).

**Total cost components**	**Values (IRR)**	**Values ($US)**
**Dialysis**	255,066,240,000	20,804,750
**Adjunct treatment**	46,706,880,000	3,809,697
**Total Costs**	301,773,120,000	24,614,447
**Total Cost per patient**	137,169,600	11,188

Considering financial, economic and quality-of-life advantages in low and middle income countries, transplantation is an attractive modality over hemodialysis (25).

*Burden of chronic kidney disease (CKD) and End-stage Renal Disease (ESRD) in Iran.*

Having introduced CKD and ESRD as public health problems, in 2004, Nafar *et al*. calculated disability adjusted life years (DALYs) for both conditions in Iran. According to their analysis, DALY for ESRD and CKD was calculated as almost 21,490 years and 1,124,164 years, respectively. It means that in Iran, more than 1 million healthy lives were lost every year due to CKD. Total DALY per 1000 population was calculated as 17.22 years, which was a considerably high value compared with other life threatening diseases such as cancers. Considering the fact that average age of the sample patient population was almost 46 years old and they were still grouped as young population, loss of productivity and decrease in active working population (as tax and insurance co- payers) are considered the most concerning issues caused by ESRD and CKD from economic point of view. Consequently, any preventive or therapy strategy which could decrease DALY or burden of ESRD and CKD in our society is strongly recommended and worth to be provided with satisfying financial resources. In this regard, screening tests to prevent ESRD as well as providing affordable RTT and maximizing patient and graft survival through providing effective immunosuppressive drugs are critical (3,25). 

## Conclusion

Renal transplantation therapy is currently fully reimbursed in Iran. However, new expensive drugs improving patient and graft survival are increasingly introduced to physicians and patients and in terms of budget limitations, making decisions about their insurance coverage is quite challenging for policy makers. Thus, estimating the current budget spending on RTT would help them in making comparison between new and current therapy strategies and would increase efficiency in resource allocation as well as decreasing current quite high out-of-pocket expenditures. 
